# Epileptic Seizure Detection Based on EEG Signals and CNN

**DOI:** 10.3389/fninf.2018.00095

**Published:** 2018-12-10

**Authors:** Mengni Zhou, Cheng Tian, Rui Cao, Bin Wang, Yan Niu, Ting Hu, Hao Guo, Jie Xiang

**Affiliations:** ^1^College of Information and Computer Science, Taiyuan University of Technology, Taiyuan, China; ^2^Software College, Taiyuan University of Technology, Taiyuan, China

**Keywords:** epilepsy, electroencephalogram, convolutional neural networks, time domain signals, frequency domain signals

## Abstract

Epilepsy is a neurological disorder that affects approximately fifty million people according to the World Health Organization. While electroencephalography (EEG) plays important roles in monitoring the brain activity of patients with epilepsy and diagnosing epilepsy, an expert is needed to analyze all EEG recordings to detect epileptic activity. This method is obviously time-consuming and tedious, and a timely and accurate diagnosis of epilepsy is essential to initiate antiepileptic drug therapy and subsequently reduce the risk of future seizures and seizure-related complications. In this study, a convolutional neural network (CNN) based on raw EEG signals instead of manual feature extraction was used to distinguish ictal, preictal, and interictal segments for epileptic seizure detection. We compared the performances of time and frequency domain signals in the detection of epileptic signals based on the intracranial Freiburg and scalp CHB-MIT databases to explore the potential of these parameters. Three types of experiments involving two binary classification problems (interictal vs. preictal and interictal vs. ictal) and one three-class problem (interictal vs. preictal vs. ictal) were conducted to explore the feasibility of this method. Using frequency domain signals in the Freiburg database, average accuracies of 96.7, 95.4, and 92.3% were obtained for the three experiments, while the average accuracies for detection in the CHB-MIT database were 95.6, 97.5, and 93% in the three experiments. Using time domain signals in the Freiburg database, the average accuracies were 91.1, 83.8, and 85.1% in the three experiments, while the signal detection accuracies in the CHB-MIT database were only 59.5, 62.3, and 47.9% in the three experiments. Based on these results, the three cases are effectively detected using frequency domain signals. However, the effective identification of the three cases using time domain signals as input samples is achieved for only some patients. Overall, the classification accuracies of frequency domain signals are significantly increased compared to time domain signals. In addition, frequency domain signals have greater potential than time domain signals for CNN applications.

## Introduction

Epilepsy, one of the most common neurological conditions characterized by epileptic seizures, is the second most common neurological disorder behind stroke, according to the World Health Organization (WHO). Seizures may occur, regardless of the circumstances or host attributes (Ahmadi et al., 2018). Patients with epilepsy suffer from sudden and unforeseen seizures, during which they are unable to protect themselves and are vulnerable to suffocation, death, or injury due to fainting and traffic accidents (Yan et al., 2016a; Mutlu, 2018). To date, this disease is mainly treated with medications and surgery; no cure exists, and treatments with anticonvulsants are not completely efficacious for all of types of epilepsy (López-Hernández et al., 2011; Yan et al., 2015).

Electroencephalography (EEG) plays an important role in detecting epilepsy, as it measures differences in voltage changes between electrodes along the subject's scalp by sense ionic currents flowing within brain neurons and provides temporal and spatial information about the brain (Misulis, 2013; Pachori and Patidar, 2014). Detection with EEG requires a direct examination by a physician as well as a substantial amount of time and effort. Furthermore, experts with differing levels of diagnostic experience sometimes report discrepant opinions on the diagnostic results (Wang et al., 2016a; Yan et al., 2017a). Therefore, the development of an automated, computer-aided method for the diagnosis of epilepsy is urgently needed (Iasemidis et al., 2005; Martis et al., 2015).

In previous studies, various detection algorithms for epileptiform EEG data have been proposed (De et al., 2008; Chen et al., 2013). Existing methods for the detection of seizures use hand-engineered techniques for feature extraction from EEG signals (Pei et al., 2018), such as time domain, frequency domain, time-frequency domain, and nonlinear signal analyses (Swapna et al., 2013; Yan et al., 2017b). After feature extraction, the selected features must be classified to recognize different EEG signals using all types of classifiers (Chen et al., 2017). Hamad et al. used the discrete wavelet transform method to extract a feature set and then trained the support vector machine (SVM) with a radial basis function, showing that the proposed gray wolf optimizer SVM approach is capable of detecting epilepsy and thus further enhancing diagnosis (Hamad et al., 2017). Subasi et al. established a hybrid model to optimize the SVM parameters based on the genetic algorithm and particle swarm optimization, showing that the proposed hybrid SVM is an efficient tool for neuroscientists to detect epileptic seizures using EEG (Subasi et al., 2017). However, these methods do not eliminate the requirement for manual feature selection (Jing et al., 2015; Wang et al., 2016b). Feature extraction is a key step in determining the classification, as it largely determines its accuracy. We boldly envision a method in which classification is performed without complex feature extraction, and the recent development of deep learning (DL) has provided a new avenue for addressing this issue.

DL has entered the mainstream in computer vision and machine learning in the last several years, exhibiting near-human and superhuman abilities to perform many tasks, such as object detection and sequence learning (Ahmedt-Aristizabal et al., 2018). Feature extraction prior to classification seems to be more preferable than directly inputting raw EEG samples into the classifier. However, in some recent studies, feature extraction was not performed, and the DL models were instead trained with raw EEG signals (Acharya et al., 2017; Hussein et al., 2018).

While most of these studies were performed based on time domain signals, some previous studies on EEG have also reported significant hidden information in the frequency domain. Wendung et al. focused on a specific category of methods based on analyses of the spatial properties of EEG signals in the time and frequency domains. These methods have been applied to both interictal and ictal recordings and share the common objective of localizing the subsets of brain structures involved in both types of paroxysmal activity (Wendung et al., 2009). Wen et al. proposed a genetic algorithm-based frequency domain feature search method that exhibited good extensibility (Wen and Zhang, 2017). Therefore, we conducted this study based on frequency domain signals and compared the seizure detection performances of both the frequency and time domains.

Here, original signals based on the time or frequency domain were directly input into the convolutional neural network (CNN) instead of extracting all feature types. We tested this method on the intracranial Freiburg database and the scalp CHB-MIT database. We not only detected binary epilepsy scenarios, e.g., interictal vs. ictal and interictal vs. preictal, but also verified the ability of this method to classify a ternary case, e.g., interictal vs. ictal vs. preictal. We compared the different performances between the time and frequency domain signals using CNN as a classifier.

This paper is organized as follows: the data, specific method proposed and performance indices are presented in the second section. Detailed experimental results are presented in the third section, and the analyses are discussed in the fourth section. The conclusions from this study are provided in the fifth section.

## Materials and Methods

### Dataset Description

One of the databases utilized in this study was prepared by the Epilepsy Center at the University Hospital of Freiburg, Germany. The database contains intracranial EEG (iEEG) data from 21 patients with medically intractable focal epilepsy that were recorded during invasive presurgical epilepsy monitoring. Intracranial grid, strip, and depth electrodes were utilized to obtain a high signal-to-noise ratio and fewer artifacts and to record directly from focal areas. The EEG data were acquired using a Neurofile NT digital video EEG system with 128 channels at a 256-Hz sampling rate (data from patient 12 were sampled at 512 Hz but downsampled to 256 Hz) (Zhang and Parhi, 2016) and a 16-bit analog-to-digital converter. All patients in the experiment had experienced 2–5 seizures, and the dataset contains recordings of 87 seizures from 21 patients. In this database, six contacts were selected for each patient by a visual inspection of the iEEG data by experienced epileptologists: three near the epileptic focus (epileptogenic zone) and three in remote locations involved in seizure spread and propagation. The subjects ranged in age from 10 to 50 years and included 13 women and 8 men. Three different seizure types were represented among the subjects, including simple partial (SP), complex partial (CP), and generalized tonic-clonic (GTC), and all subjects had experienced at least two types. The epileptic focus was located in neocortical brain structures in eleven patients, in the hippocampus in eight patients, and in both locations in two patients. The seizure onset times and epileptiform activities were annotated by certified epileptologists at the Epilepsy Center.

The other database used in this study was an open-source EEG database from CHB-MIT (http://physionet.org/cgi-bin/atm/ATM). The recordings were collected from 23 children with epilepsy using scalp electrodes, and EEG data were provided by the Massachusetts Institute of Technology (MIT, USA). The study included 17 females that ranged in age from ~1.5 to 19 years and five males that ranged in age from 3 to 22 years. The age and sex information for one child was lost. All subjects were asked to stop related treatments 1 week before data collection. The sampling frequency for all patients was 256 Hz. The seizure start and end times were labeled explicitly based on expert judgments, and the number and durations of seizure events varied for each subject.

For the detection of ictal, preictal and interictal signals, many segments were chosen for these two open-source databases. The period when patients experience seizure onset is named the ictal state and is easily detected from raw signals by experts. The interictal period corresponds to the normal state between two seizures. The transition from the interictal period to the ictal period is the preictal period. In this study, the differences were evaluated by applying the CNN to each patient, and the moving-window technique was employed to divide raw recordings into 1-s epochs.

### Time and Frequency Domain Signals

In the present study, we used time or frequency domain signals as inputs for classification. The frequency domain is a coordinate system that describes the frequency features of the signals. A frequency spectrogram reflects the relationship between the frequency and amplitude of a signal and is often used to analyze signal features (Wen and Zhang, 2017). For each channel, we first converted the time domain signals into frequency domain signals using the fast Fourier transform (FFT) method (Rasekhi et al., 2013).

**Figure 1:**
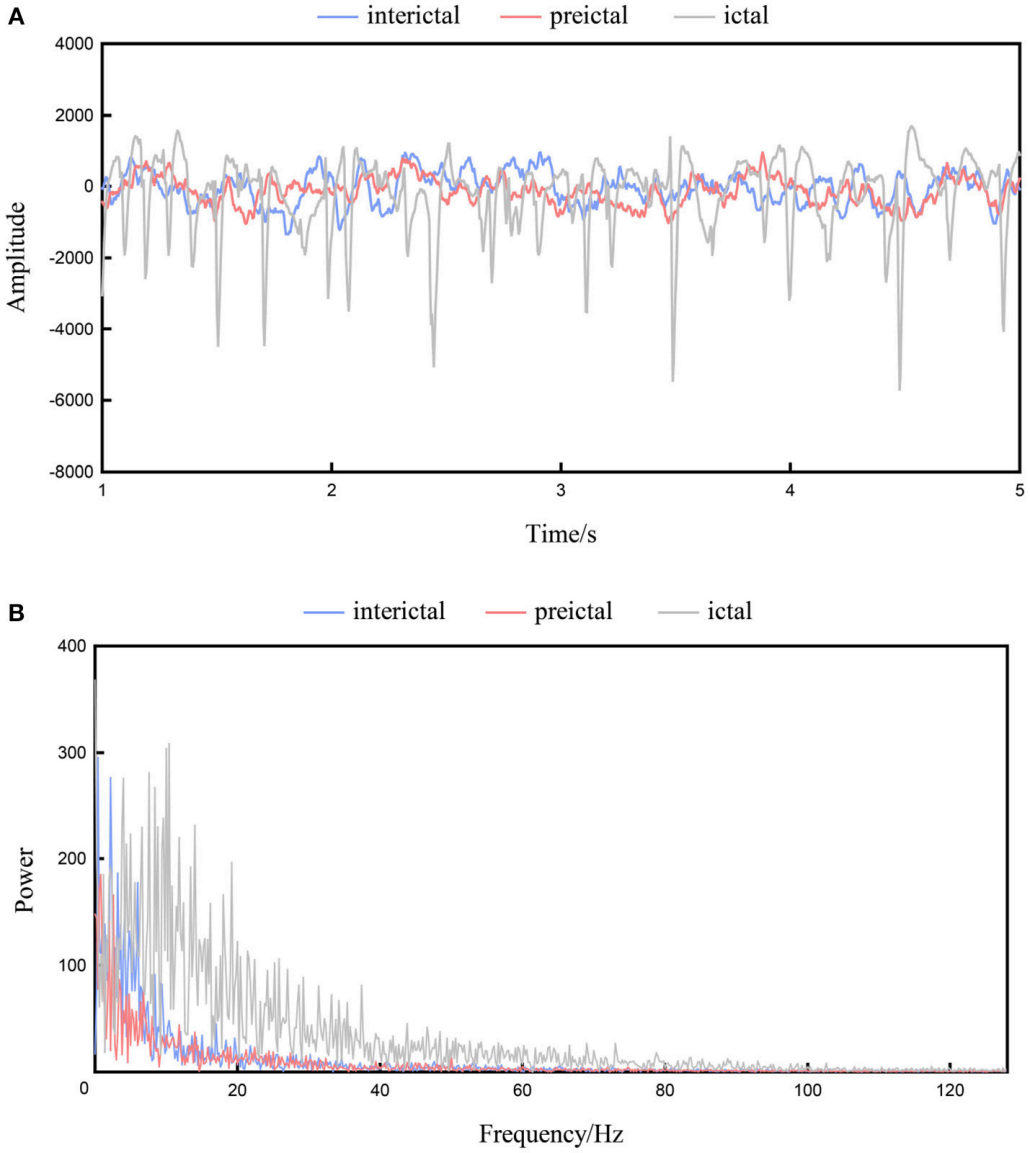
The interictal, preictal and ictal recordings from patient 1. **(A)** Recordings of the time domain. **(B)** Recordings of the frequency domain.

Figure 1A shows the interictal, preictal, and ictal recordings of a channel from the time domain of patient 3 in the Freiburg database. The EEG signal is obviously nonlinear and nonstationary in nature, while the signal is highly complex, and a visual interpretation of the signals is difficult (Acharya et al., 2017). Figure 1B shows the frequency domain signals resulting from the application of FFT to the interictal, preictal, and ictal recordings shown in Figure 1A. The x-axis represents the frequency, whereas the y-axis represents the amplitude. Significant variations are observed among the ictal, preictal, and interictal signals at certain frequencies, and these features are suitable for classification. In contrast, the amplitudes at some other frequencies are difficult to distinguish, and these enclosed features are ineffective. Classifiers require a number of effective features. Compared with time domain signals, frequency domain signals are more obvious in EEG data (Ren and Wu, 2014).

### CNN

The use of CNNs for large-scale imaging and video recognition has been very successful (Sermanet et al., 2013; Simonyan and Zisserman, 2014a) due to the establishment of large public image repositories, such as ImageNet (Deng et al., 2009), and high-performance computing systems, such as large-scale distributed clusters (Dean et al., 2012; Simonyan and Zisserman, 2014b). Recently, some studies have begun applying CNNs to EEG signals (Ullah et al., 2018), and research interest in using CNNs for seizure prediction has increased, probably because these methods have been used extensively and are thus better established and more familiar in the research community.

A CNN consists of an input and an output layer, as well as multiple hidden layers. The hidden layers of a CNN typically consist of convolutional layers, pooling layers and fully connected layers. Convolutional layers apply a convolution operation to the input, transferring the result to the next layer. The convolution emulates the response of an individual neuron to visual stimuli. Convolutional networks may include local or global pooling layers that combine the outputs of neuron clusters in one layer into a single neuron in the next layer. Mean pooling uses the average value from each cluster of neurons in the previous layer. Fully connected layers connect every neuron in one layer to every neuron in another layer. The CNN is in principle the same as the traditional multi-layer perceptron neural network.

Compared with traditional classifiers, CNNs have obvious advantages for analyzing high-dimensional data. CNNs employ a parameter sharing scheme, which is used in convolutional layers to control and reduce the number of parameters. A pooling layer is designed to progressively reduce the spatial size of the representation and the number of parameters and computation in the network, and subsequently control overfitting.

**Figure 2:**
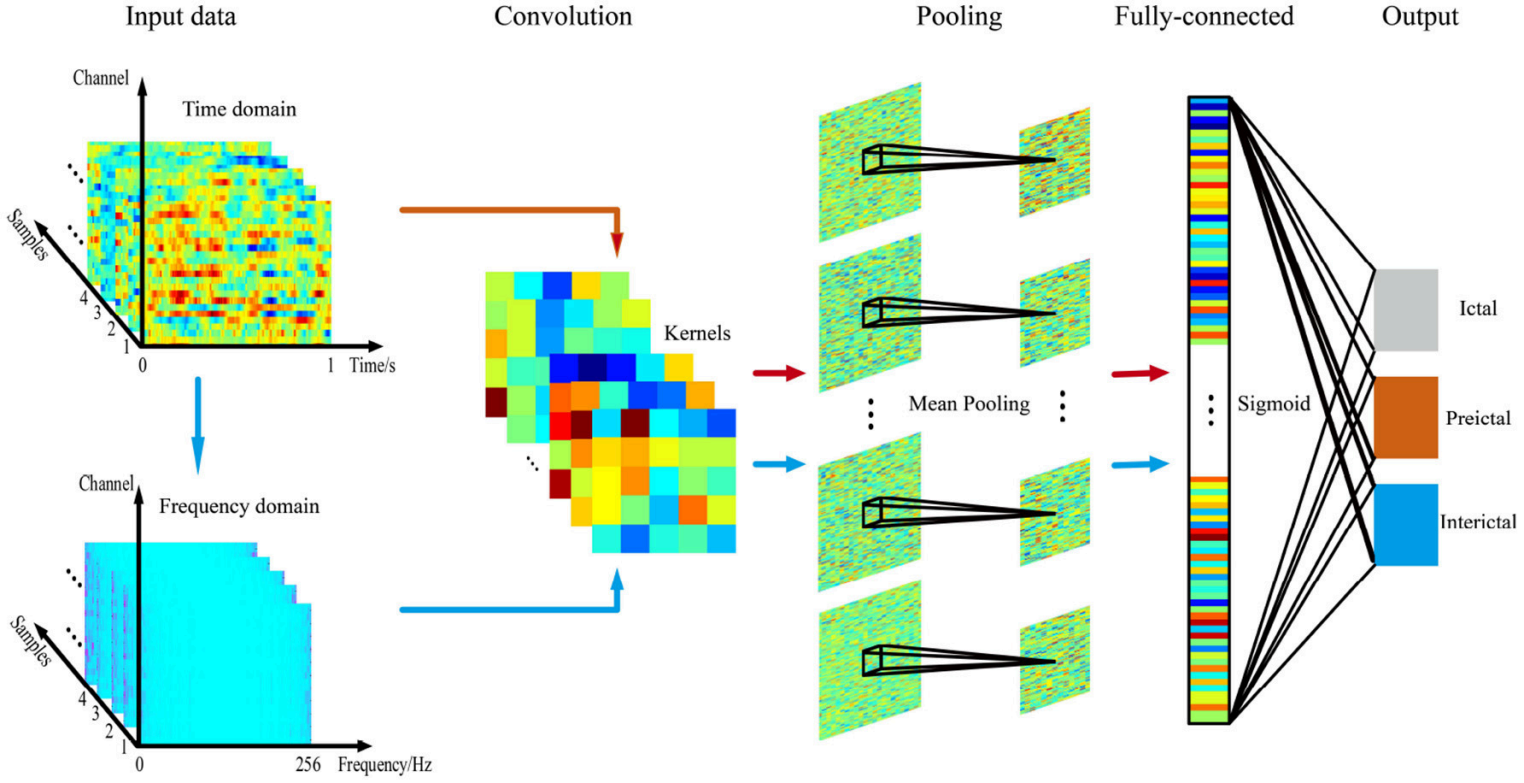
Illustration of the CNN.

As shown in Figure 2, a multichannel time series based on time or frequency domain signals was directly input into a CNN as the input layer. The CNN models we used consisted of three main layers. Structurally, CNNs have convolutional layers interspersed with pooling layers, followed by fully connected layers. The convolutional layer, which has 6 feature maps connected to the input layer via 5^*^5 kernels, consists of kernels that slide across the EEG signals. A kernel comprises the matrix to be convolved with the input EEG signal and stride (stride = 1) and controls the extent to which the filter convolves across the input signal. The second layer comprises a 2^*^2 mean pooling layer and is mainly used to extract key features and reduce the computational complexity of the network. The final fully connected layer outputs the classification result (i.e., ictal, preictal, or interictal) using sigmoid activation.

In this study, we designed a CNN with no more than three layers for multiple reasons. On one hand, the number of samples acquired during ictal and preictal recordings is usually much smaller than the number acquired during the interictal period in the epilepsy database, leading to a serious imbalance in the number of samples, and a simple structure meets the demand for fewer samples. In addition, the small number of electrodes also limits the number of layers in the network to some extent. On the other hand, a simple training structure is more conducive to the online clinical diagnosis of epileptic signals (Yan et al., 2018).

**Figure 3:**
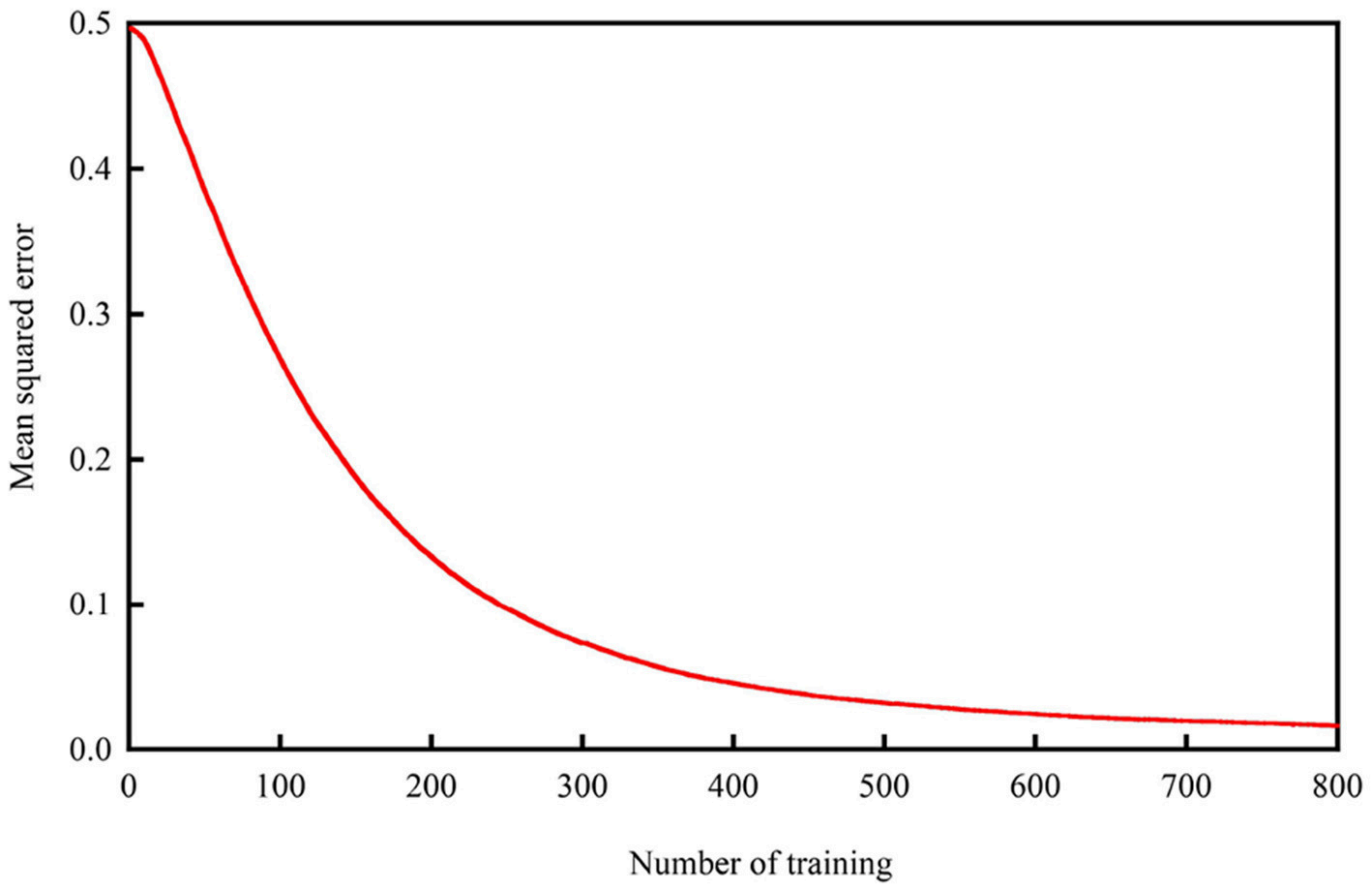
Mean squared error.

The detection system was tested on all patients. The dataset was further randomly partitioned into training and independent testing sets via 6-fold cross validation to ensure that the results were valid and generalizable for making predictions from new data. Each of the six subsets acts as an independent holdout test set for the model trained with the remaining five subsets (Xiang et al., 2015). During each run, five subsets are used for training, and the remaining subset is used for testing, providing the advantage that all test sets are independent of one another (Kevric and Subasi, 2014). Numerous trials were performed to test which of the internal architectures analyzed in our experiment provided the most reasonable and proper results until the mean squared error curve normalized, as shown in Figure 3.

### Prediction of Performance Indices

The statistical measures for assessing the classification performance included accuracy (acc), sensitivity (sen) and specificity (spe), which were calculated as follows:

(1) $$\begin{array}{l} sen=\frac{TP}{TP+FN} \end{array}$$

(2) $$\begin{array}{l} spe=\frac{TN}{FP+TN} \end{array}$$

(3) $$\begin{array}{l} acc=\frac{TP+TN}{P+N} \end{array}$$

*P* denotes the number of samples during a preictal or ictal period, *N* denotes the number of samples during an interictal period, *FP* denotes the number of samples in an interictal period that were mistaken for a preictal or ictal period, *FN* denotes the number of samples in a preictal or ictal period that were mistaken for an interictal period, and *TP* and *TN* denote the numbers of samples that were accurately classified. These three measures were used to evaluate the performance of the method to assess binary classification problems. For three-class problems, only accuracy was considered.

## Results

The methodology described here was evaluated using the Freiburg and CHB-MIT databases based on time and frequency domain signals. This system was tested on three cases: two types of experiments involving binary classification problems [(i) interictal vs. preictal and (ii) interictal vs. ictal] and one three-class problem (interictal vs. ictal vs. preictal). We trained and tested our method for each patient individually, and the classification results for all patients analyzed are presented in Table 1 through **Table 4**. The average accuracy, sensitivity and specificity values obtained are also indicated.

**Table 1:** Frequency domain signal results for all patients in the Freiburg database.

**Patient ID**	**Binary Case**						**Interictal vs. Ictal vs. Preictal**
	**Interictal vs. Preictal**			**Interictal vs. Ictal**			
	**acc**	**sen**	**spe**	**acc**	**sen**	**spe**	**acc**
1	0.967	0.960	0.973	0.960	0.940	0.980	0.930
2	0.997	0.997	0.997	0.975	0.957	0.993	0.963
3	0.945	0.960	0.930	0.945	0.933	0.957	0.928
4	0.995	1.000	0.990	0.992	0.997	0.987	0.986
5	0.982	0.983	0.980	0.988	0.987	0.990	0.971
6	0.995	1.000	0.990	0.988	0.997	0.980	0.911
7	0.980	0.987	0.973	0.968	0.943	0.993	0.937
8	0.815	0.833	0.797	0.755	0.743	0.767	0.678
9	1.000	1.000	1.000	1.000	1.000	1.000	0.913
10	0.992	0.987	0.997	0.967	0.943	0.990	0.967
11	1.000	1.000	1.000	0.973	0.947	1.000	0.963
12	0.993	0.987	1.000	0.987	0.973	1.000	0.986
13	1.000	1.000	1.000	0.970	0.957	0.983	0.896
14	1.000	1.000	1.000	0.998	0.997	1.000	0.954
15	0.958	0.953	0.963	0.902	0.857	0.947	0.856
16	0.868	0.860	0.877	0.907	0.860	0.953	0.867
17	0.958	0.943	0.973	0.987	0.983	0.990	0.956
18	0.943	0.927	0.960	0.920	0.890	0.950	0.914
19	0.965	0.963	0.967	0.948	0.903	0.993	0.960
20	0.960	0.960	0.960	0.925	0.870	0.980	0.920
21	1.000	1.000	1.000	0.985	0.990	0.980	0.933
Avg	0.967	0.967	0.968	0.954	0.937	0.972	0.923

### Results From the Freiburg Database

#### Results for the Frequency Domain Signals

The experimental results of the segment-based performance assessment of this method for patients in the Freiburg database are listed in Table 1. The detection quality obviously varied with the subjects due to the individual differences in humans. The final row of Table 1 displays the average results of the three statistical measures (accuracy, sensitivity, and specificity) for all 21 patients.

The mean accuracy of classification between the interictal and preictal signals was 96.7%, and the average sensitivity and specificity values were 96.7 and 96.8%, respectively. The best classification results were observed for patients 9, 11, 13, 14, and 21, while some patients had poor results, such as patient 8. The sensitivity and specificity values for this patient were very unsatisfactory—at 83.3 and 79.7%, respectively. Overall, the accuracy of classification was >90% for nearly all the patients, except for patients 8 and 16. The classification sensitivity and specificity values for these patients were relatively balanced.

Good results were also obtained for classification between interictal and ictal signals, as this method exhibited average accuracy, sensitivity, and specificity values of 95.4, 93.7, and 97.2%, respectively. The classification accuracy for patient 8 was less than 90%, while this value was >90% for all other patients. The binary classification of signals from patient 9 remained satisfactory. The results presented in the table show that the classification sensitivities and specificities for each patient were clearly balanced.

For the classification of interictal, ictal, and preictal signals, only the accuracy of every patient is presented; the average accuracy of classification among the 21 patients was 92.3%. Among these patients, the accuracies of classification for nine patients were >95%, which was considered a great result, and the classification accuracies were good for eight patients, with values ranging between 90 and 95%. The accuracy of signal classification for the other four patients was <90%.

#### Results for the Time Domain Signals

Table 2 reports the classification results for time domain signals from patients in the Freiburg database. The average accuracies of the three experiments were 91.1, 83.8, and 85.1%, respectively.

**Table 2:** Time domain signal results for all patients in the Freiburg database.

**Patient ID**	**Binary Case**						**Interictal vs. Ictal vs. Preictal**
	**Interictal vs. Preictal**			**Interictal vs. Ictal**			
	**acc**	**sen**	**spe**	**acc**	**sen**	**spe**	**acc**
1	0.968	0.973	0.963	0.857	0.723	0.990	0.961
2	0.997	0.997	0.997	0.880	0.797	0.963	0.964
3	0.997	0.993	1.000	0.907	0.900	0.913	0.973
4	0.968	0.983	0.953	0.913	0.947	0.880	0.956
5	0.977	0.963	0.990	0.953	0.963	0.943	0.971
6	0.987	0.983	0.990	0.920	0.860	0.980	0.900
7	0.888	0.873	0.903	0.858	0.797	0.920	0.860
8	0.580	0.507	0.653	0.598	0.550	0.647	0.488
9	0.737	0.743	0.730	0.825	0.793	0.857	0.530
10	0.932	0.930	0.933	0.850	0.903	0.797	0.922
11	0.677	0.677	0.677	0.675	0.647	0.703	0.616
12	0.997	0.997	0.997	0.833	0.773	0.893	0.981
13	0.720	0.770	0.670	0.725	0.587	0.863	0.610
14	0.955	0.943	0.967	0.985	0.993	0.977	0.953
15	0.995	0.993	0.997	0.633	0.603	0.663	0.628
16	0.973	0.963	0.983	0.813	0.813	0.813	0.931
17	0.872	0.893	0.850	0.792	0.887	0.697	0.853
18	0.983	0.980	0.987	0.842	0.790	0.893	0.940
19	0.997	0.993	1.000	0.832	0.673	0.990	0.964
20	0.988	1.000	0.977	0.980	0.987	0.973	0.949
21	0.938	0.977	0.900	0.917	0.900	0.933	0.921
Avg	0.911	0.911	0.910	0.838	0.804	0.871	0.851

For interictal vs. preictal signals, the averages of three measures were >90% for all patients. However, unsatisfactory results for either accuracy, sensitivity or specificity values were obtained for six patients. Almost ideal results were obtained for some individuals, such as patients 2, 3, 15, and 19.

When classifying the interictal and ictal segments, the overall results were slightly worse, as values of only 83.8, 80.4, and 87.1% were obtained for the three measures, respectively. An accuracy of >90% was achieved for only seven patients, and the accuracy of classifying signals from patient 8 was <60%. Accuracies between 60 and 70% were obtained for patients 11 and 15, and the accuracies of classifying signals from all other patients was generally good.

For the three-class problem, the average accuracy was 85.1%, and a 90% classification accuracy was reported for 67% of the patients, with the highest value reaching 98.1%. Notably, the accuracy of signal classification in some patients, such as patients 8 and 9, was very unsatisfactory.

#### Comparison of the Frequency and Time Domains

**Figure 4:**
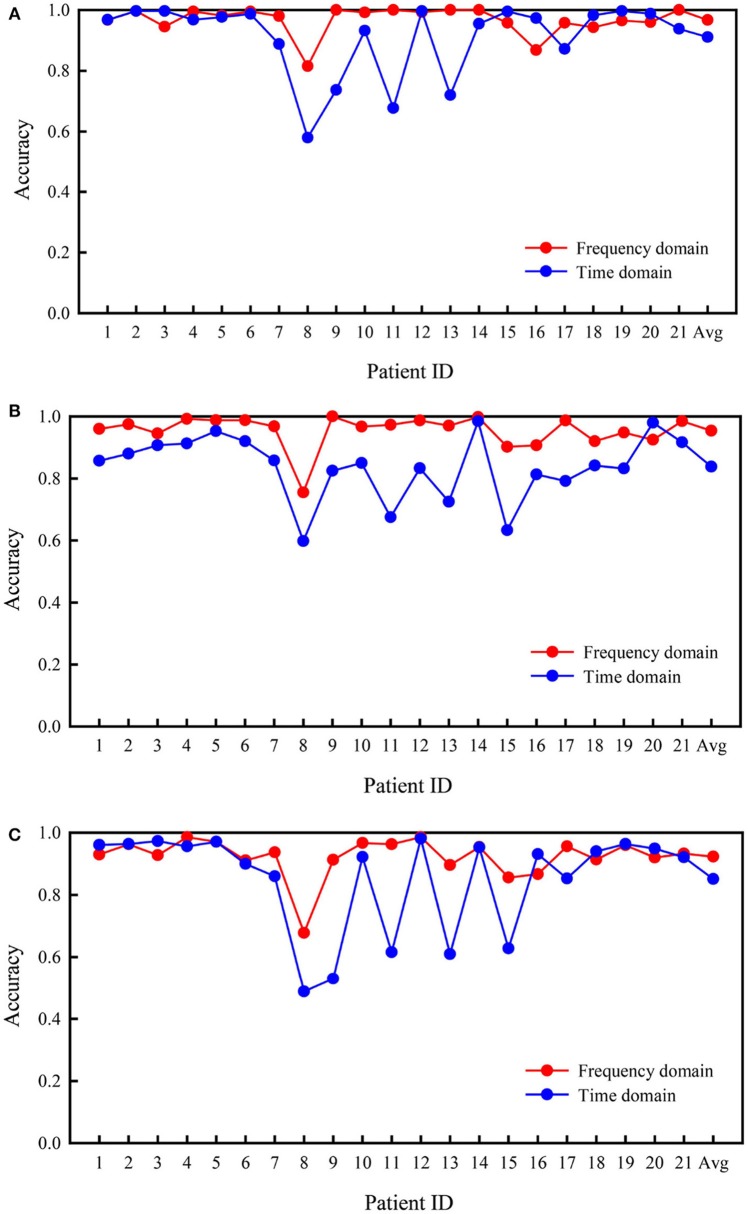
Comparison of accuracies in the Freiburg database based on frequency and time domain signals. **(A)** Interictal vs. preictal. **(B)** Interictal vs. ictal. **(C)** Interictal vs. ictal vs. preictal.

Figure 4 presents the comparison of accuracy values based on the time and frequency domains for all patients in the two types of binary classification problems and the three-class problem. In the three experiments, the average accuracies of the frequency domain were higher than those of the time domain. As shown in Figure 4A, better results were obtained for the classification of interictal vs. preictal signals using the time domain than the frequency domain in some patients, while the opposite trend was observed in the other patients. The results were far worse using the time domain than the frequency domain for several patients, such as patients 8 and 9. As depicted in Figure 4B, the frequency domain results were better at classifying the interictal vs. ictal signals than the time domain results for all patients, except patient 20. The frequency and time domain results were very similar for patient 14. The results shown in Figure 4C are similar to those shown in Figure 4A; however, the average performance of the frequency domain was higher than the time domain.

### Results From the CHB-MIT Database

#### Results for the Frequency Domain Signals

Table 3 shows the results for all patients in the CHB-MIT database based on frequency domain signals. Similar to the Freiburg database, three different experiments were conducted using patients from this database.

**Table 3:** Frequency domain signal results for all patients in the CHB-MIT database.

**Patient ID**	**Binary Case**						**Interictal vs. Ictal vs. Preictal**
	**Interictal vs. Preictal**			**Interictal vs. Ictal**			
	**acc**	**sen**	**spe**	**acc**	**sen**	**spe**	**acc**
1	0.992	0.987	0.997	0.983	0.983	0.983	0.987
2	0.917	0.853	0.980	0.972	0.967	0.977	0.937
3	0.978	0.993	0.963	0.977	0.983	0.970	0.978
4	0.922	0.893	0.950	0.982	0.987	0.977	0.953
5	0.985	0.980	0.990	0.980	0.980	0.980	0.971
6	0.958	0.963	0.953	0.998	1.000	0.997	0.976
7	0.977	0.973	0.980	1.000	1.000	1.000	0.972
8	1.000	1.000	1.000	0.993	0.990	0.997	0.978
9	0.998	0.997	1.000	1.000	1.000	1.000	0.984
10	0.920	0.900	0.940	0.960	0.943	0.977	0.909
11	0.982	0.977	0.987	0.980	0.967	0.993	0.949
12	0.953	0.910	0.997	0.992	0.983	1.000	0.939
13	0.970	0.943	0.997	0.975	0.960	0.990	0.969
14	0.942	0.950	0.933	0.915	0.873	0.957	0.701
15	0.968	0.937	1.000	0.983	0.970	0.997	0.970
16	0.928	0.920	0.937	0.962	0.947	0.977	0.898
17	0.898	0.853	0.943	0.967	0.963	0.970	0.903
18	0.968	0.937	1.000	0.985	0.993	0.977	0.927
19	0.998	0.997	1.000	0.988	0.987	0.990	0.974
20	1.000	1.000	1.000	0.985	0.990	0.980	0.984
21	0.840	0.840	0.840	0.925	0.893	0.957	0.810
22	0.925	0.900	0.950	0.970	0.950	0.990	0.939
23	0.928	0.927	0.930	0.982	1.000	0.963	0.823
24	0.988	0.987	0.990	0.947	0.937	0.957	0.894
Avg	0.956	0.942	0.969	0.975	0.969	0.981	0.930

For the classification of interictal and preictal signals, the average accuracy, sensitivity and specificity of results obtained using this database were 95.6, 94.2, and 96.9%, respectively. The best results for the three measures were obtained from patients 8 and 20, while the classification accuracy was unsatisfactory (84.0%) for some patients, such as patient 21. The classification accuracy for patient 17 was <90%. Overall, the accuracy, sensitivity and specificity values of classification were >90% for most patients.

When applying this method to the classification of interictal and ictal signals, the average values of the three measures were >90% for all patients, and the average results were better than the classification of interictal and preictal signals. From the overall perspective of all patients, the sensitivities of classification for patients 14 and 21 were <90% but >85%. All other values of the three measures were >90%.

For the three-class problem, an accuracy of 93.0% was obtained, and the classification results for some patients, such as patients 1 and 9, were very good. A poor accuracy of signal classification was observed only for patient 14. The accuracy of signal classification for four patients (patients 16, 21, 23, and 24) was unsatisfactory, ranging from 80 to 90%, while the accuracy of signal classification for the other patients was >90%.

#### Results for the Time Domain Signals

Table 4 shows the time domain signal data for all patients in the CHB-MIT database. The average performances of the three experiments were obviously poor, with average accuracies of 59.5, 62.3, and 47.9%, respectively. A good result was obtained in the three experiments for only one patient, while the results for all other patients were disappointing. The diagnostic performances of classifying interictal vs. preictal signals in some patients, such as patients 4 and 5, were maintained at only a random level, and the results obtained for patients 22 and 23 were very poor and below random levels. The average accuracy of classification of interictal and ictal segments was slightly better than the classification of interictal and preictal signals. Inevitably, the accuracy of classification for individual subjects was maintained at only random or lower than random levels. The average accuracy of classifying interictal vs. ictal vs. preictal signals was 47.9%.

**Table 4:** Time domain signal results for all patients in the CHB-MIT database.

**Patient ID**	**Binary Case**						**Interictal vs. Ictal vs. Preictal**
	**Interictal vs. Preictal**			**Interictal vs. Ictal**			
	**acc**	**sen**	**spe**	**acc**	**sen**	**spe**	**acc**
1	0.905	0.900	0.910	0.993	0.987	1.000	0.934
2	0.623	0.607	0.640	0.475	0.450	0.500	0.419
3	0.623	0.677	0.570	0.677	0.713	0.640	0.460
4	0.515	0.573	0.457	0.522	0.477	0.567	0.342
5	0.508	0.533	0.483	0.568	0.520	0.617	0.409
6	0.530	0.547	0.513	0.557	0.450	0.663	0.370
7	0.627	0.660	0.593	0.717	0.677	0.757	0.473
8	0.508	0.547	0.470	0.548	0.580	0.517	0.378
9	0.538	0.563	0.513	0.698	0.667	0.730	0.459
10	0.620	0.637	0.603	0.508	0.493	0.523	0.361
11	0.548	0.607	0.490	0.550	0.540	0.560	0.410
12	0.820	0.860	0.780	0.712	0.707	0.717	0.581
13	0.582	0.630	0.533	0.525	0.600	0.450	0.498
14	0.503	0.513	0.493	0.650	0.670	0.630	0.453
15	0.605	0.580	0.630	0.578	0.580	0.577	0.463
16	0.507	0.523	0.490	0.537	0.433	0.640	0.384
17	0.505	0.480	0.530	0.692	0.643	0.740	0.473
18	0.740	0.810	0.670	0.628	0.667	0.590	0.480
19	0.745	0.700	0.790	0.602	0.507	0.697	0.679
20	0.590	0.657	0.523	0.490	0.553	0.427	0.514
21	0.572	0.587	0.557	0.545	0.560	0.530	0.386
22	0.462	0.477	0.447	0.500	0.530	0.470	0.369
23	0.492	0.517	0.467	0.762	0.777	0.747	0.502
24	0.615	0.650	0.580	0.915	0.917	0.913	0.690
Avg	0.595	0.618	0.572	0.623	0.612	0.633	0.479

#### Comparison of the Frequency and Time Domains

**Figure 5:**
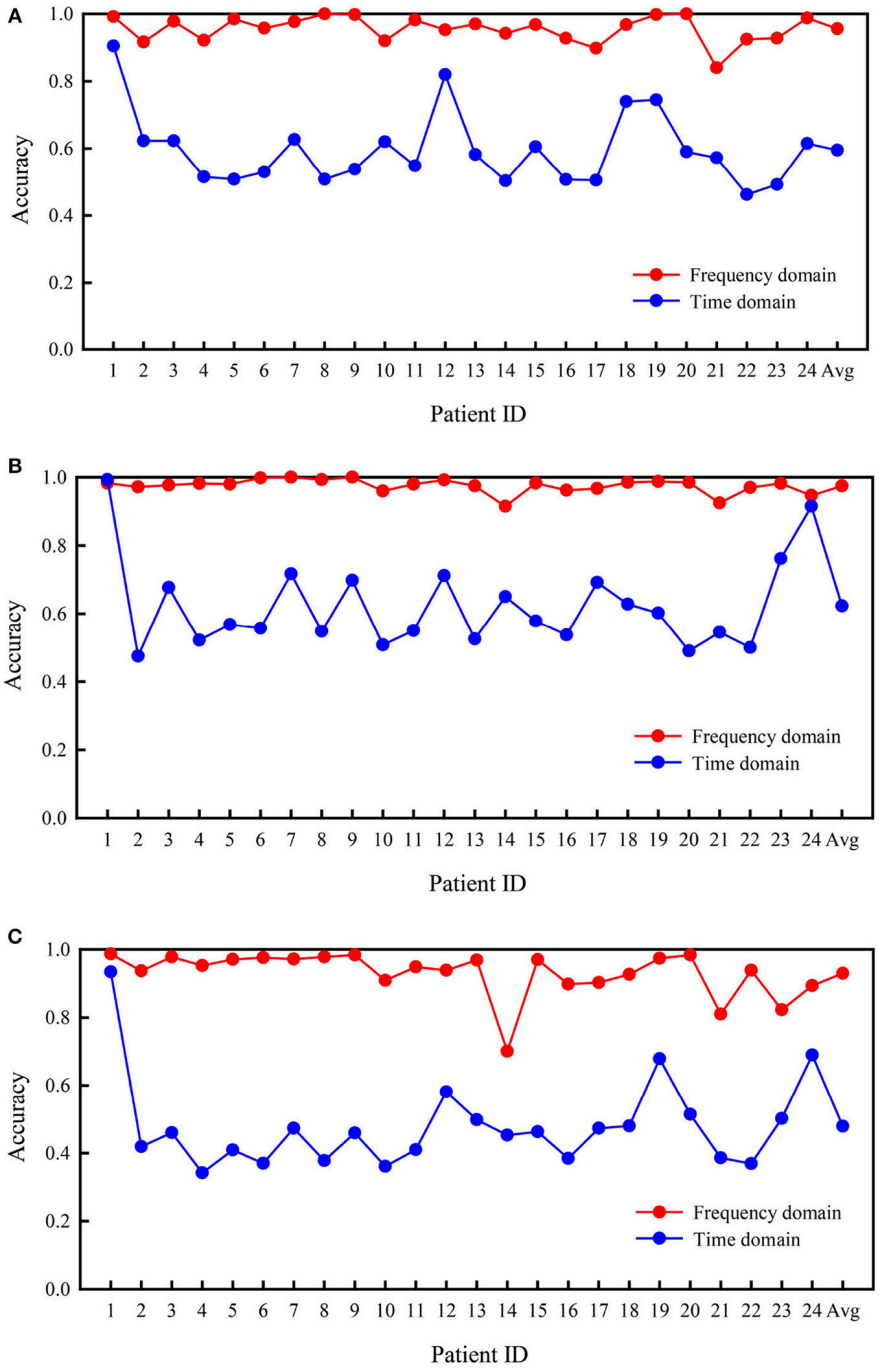
Comparison of accuracies based on frequency and time domain signals from subjects in the CHB-MIT database. **(A)** Interictal vs. preictal. **(B)** Interictal vs. ictal. **(C)** Interictal vs. ictal vs. preictal.

Figure 5 summarizes the comparison of the classification performances based on frequency and time domain signals from subjects in the CHB-MIT database. Generally, the three cases were detected effectively using frequency domain signals. The classification based on the frequency domain was remarkably more accurate than classification based on the time domain. The mean accuracies of classification calculated using frequency domain signals were 95.6, 97.5, and 93.0% for the three experiments, which were significantly greater than values calculated using time domain signals (59.5, 62.3, and 47.9%, respectively). The classification performances calculated using the frequency domain were higher than those calculated using the time domain signals for all patients.

## Discussion

### Comparison With Other Methods

Many other methods for detecting epileptic seizures have been proposed by other researchers. For example, Shoeb and Guttag presented a patient-specific machine learning technique based on the CHB-MIT database. They extracted spectral and spatial features and then combined non-EEG features to form a feature vector; an SVM was then used for classification. Their approach detected 96% of 173 test seizures in an event-based assessment (Shoeb and Guttag, 2010). A method based on the Freiburg database was presented in another study (Patnaik and Manyam, 2008) in which the authors used wavelet transform and neural networks together with the application of harmonic weight for classification; this method presented an average specificity and sensitivity of 99.19 and 91.29%, respectively. Another patient-specific seizure detection method using the Freiburg database has been described (Yuan et al., 2012). The fractal intercept derived from fractal geometry was extracted as a novel nonlinear feature of EEG signals, and the relative fluctuation index was calculated as a linear feature. The feature vector consisting of the two EEG descriptors was fed into a single-layer neural network for classification. For the segment-based level, the sensitivity was 91.72%, and the specificity was 94.89%. These existing methods for the detection of seizures use hand-engineered techniques to extract features from EEG signals. Their performance strongly depends on the selection of hyperparameters and the data, and research requires not only a wealth of expertise but also a substantial amount of labor. Therefore, automatic feature learning has a substantial advantage over the traditional methods of manual feature extraction (Ullah et al., 2018). CNNs are a type of a DL method that processes data without requiring manual feature extraction or selection. CNNs extract features more discriminatively and robustly than hand-designed features and adapt to internal data structures (Cun, 1995).

Of course, some studies have used DL for seizure detection. A 13-layer deep CNN algorithm was implemented to detect normal, preictal and seizure classes using the Bonn database (Acharya et al., 2017). The proposed technique exhibited accuracy, specificity and sensitivity values of 88.67, 90.00, and 95%, respectively, but the 13-layer deep CNN may obviously require a substantial amount of labor to elucidate the best network structure. In our study, the CNN included only three main layers, and the network was very simple compared with the deep network. Meanwhile, satisfactory results were obtained from both databases analyzed using the same network. In addition, a 1-s time segment was used for detection once the model was completely trained. All of these features provide great possibilities for real-time detection in the clinic.

Compared with the studies described above, our study reported equal or even better performance. For the Freiburg database, we obtained average accuracies of 96.7, 95.4, and 94.3% for all three experiments, while the average accuracies obtained using the CHB-MIT database were 95.6, 97.5, and 93% for the three cases analyzed. In the present study, we analyzed two types of binary classification problems and a three-class problem using both intracranial data and scalp data based on the proposed method. Three-class problems have rarely been tested using data from these two databases and achieved good results, and a large number of results will be powerful for proving the feasibility of the method.

### Frequency and Time Domains

Many existing automatic seizure detection techniques use traditional signal processing and machine learning techniques. Some of these techniques show good accuracy for one problem but fail to perform accurately for others, e.g., they classify seizure vs. nonseizure cases with good accuracy but show poor performance for distinguishing normal vs. ictal vs. interictal signals (Zhang et al., 2017). One of the remaining challenges is the development of a generalized model that classifies both binary and ternary problems. Therefore, we tested this system on three cases: (i) interictal vs. preictal, (ii) interictal vs. ictal and (iii) interictal vs. ictal vs. preictal. The results obtained from all three experiments exhibited >90% accuracy, even for the ternary problem based on the frequency domain, although the performance of the system for classifying the ternary problem was decreased to a certain degree. For all three cases, the frequency domain performed better than the time domain.

In addition, one challenge underlying the development of a successful seizure detection method is that some methods exhibit excellent results based on their own databases, but their performance decreases when other databases are used. Thus, the identification of a method that adequately adapts to multiple datasets is challenging. Furthermore, the characteristics of EEG analyses of different brain locations, patient ages, patient sexes and seizure types vary significantly among patients with epilepsy, leading to substantial individual differences (Wilson et al., 2004; Yang et al., 2018). In this study, we used two completely different databases to test related methods, and the patients in these two databases exhibited several types of seizures and large age ranges. According to our results, the average accuracy of results based on the frequency domain was better than results based on the time domain in all experiments, regardless of whether the Freiburg or CHB-MIT database was used. In addition, better results were obtained for most patients when the frequency domain was analyzed. Therefore, this method might be adapted to account for individual differences or other epileptic databases to a certain extent. The accuracy range was smaller in the frequency domain than in the time domain across all patients in both databases. Therefore, individual differences may have less of an impact on the performance of the method based on the frequency domain than on the time domain, indicating greater stability.

Finally, seizure detection is challenging because the electrical activity of the brain is mediated by numerous classes of neurons with overlapping characteristics (Shoeb and Guttag, 2010), and improvements in the detection performance by extracting more effective features and excluding irrelevant features or redundant features among different classes is thus impossible. In our study of the Freiburg database, the performance of the time domain was better than the frequency domain for some patients, but the average performance of the frequency domain was still better. For the CHB-MIT database, the frequency domain performed better than the time domain in almost all situations. In other words, both the two-class and three-class signals were effectively detected using frequency domain signals. The classification based on the frequency domain was remarkably more accurate, sensitive and specific than classification based on the time domain for both databases. Therefore, the CNN may more easily extract more effective features based on the frequency domain than on the time domain.

### Impacts of the Two Databases

We completed three sets of experiments using two different public databases. For the analysis of frequency domain signals in the Freiburg database, average accuracies of 96.7, 95.4, and 92.3% were obtained for the three experiments. For the CHB-MIT database, the average accuracies of the three experiments were 95.6, 97.5, and 93%. Comparable performances were observed in these two datasets when frequency domain segments were used as input samples. However, the two sets of data showed significant differences when the original signal was used as the training data. For the Freiburg database, the average accuracies were 91.1, 83.8, and 85.1% in the three experiments, while the average accuracies for the CHB-MIT database were only 59.5, 62.3, and 47.9%. One potential explanation for this discrepancy is that the data in the Freiburg database were obtained from intracranial signals, while the signals in the CHB-MIT database were obtained from scalp electrodes. Intracranial signals have a high signal-to-noise ratio and few artifacts, while signals from scalp electrodes contain more noise interference, which may result in the extraction of low-quality features. Another potential explanation for this discrepancy is that the signals in the Freiburg database were recorded directly from focal areas, while signals in the CHB-MIT database were recorded from whole-brain electrodes, and more redundant information may have been included. Intracranial EEGs also include features that are not observed within the scalp EEGs because of the spatial averaging effect of the dura and skull (Shoeb and Guttag, 2010).

## Conclusions

Currently, epileptic activity in EEG recordings is mainly examined using a number of traditional and trending technologies. Automation of this process presents many advantages, including a faster diagnosis, continuous monitoring, and reduction in the overall cost of medical treatment (Yan et al., 2016b). We conducted experiments to compare the performances of time and frequency domain signals. The method not only avoided the complex feature extraction process but also used a very simple CNN structure. Both the Freiburg and CHB-MIT datasets were analyzed to confirm the validity of our method, and frequency domain signals performed better than time domain signals. When frequency domain signals were analyzed, both two- and three-class problems were solved with satisfactory results. One limitation of this study is that the large volumes of continuous EEG recordings required for deep learning algorithms are limited. In addition, the non-abruptness phenomenon and inconsistency of the signals, along with different brain location, patient ages, patient sexes and seizure types are challenging issues that affect the consistency of performance. In the future, we plan to apply this method to online epileptic signal detection. After classification, our next research object is to develop a successful seizure forecasting model.

## Author Contributions

MZ completed the entire study of the experiment and writing. CT, RC, and BW provided advice and guidance. YN, TH, and HG revised the manuscript. JX provided the research ideas.

### Conflict of interest statement

The authors declare that the research was conducted in the absence of any commercial or financial relationships that could be construed as a potential conflict of interest.
